# Telehealth Use among Community Health Centers and Cardio-Metabolic Health Outcomes

**DOI:** 10.3390/healthcare8020165

**Published:** 2020-06-10

**Authors:** Navneet Kaur Baidwan, Ganisher Davlyatov, Tapan Mehta

**Affiliations:** 1 UAB/Lakeshore Research Collaborative, University of Alabama at Birmingham, Birmingham, AL 35294, USA; gani@uab.edu (G.D.); tapan@uab.edu (T.M.); 2 Department of Health Services Administration, School of Health Professions, University of Alabama at Birmingham, Birmingham, AL 35294, USA; 3 Nutrition Obesity Research Center, University of Alabama at Birmingham, Birmingham, AL 35294, USA

**Keywords:** telehealth, community health centers, cardio-metabolic outcomes

## Abstract

Public health interventions to manage the cardio-metabolic syndrome (CMS) have had modest success, necessitating the expansion of telehealth services to where people live. This effort analyzes the association between telehealth provision and the management of CMS-related quality measures (hypertension, diabetes, weight assessment and related counseling, lipid therapy for coronary artery disease, and antiplatelet therapy for ischemic vascular disease) using the Uniform Data System administrative database during the period 2016–2018. A total of 523, 600, and 586 community health centers (CHCs) were documented using telehealth, out of the 1367, 1373, and 1362 total CHCs, in the respective three years. Our primary analysis showed that there was no association between telehealth use and the outcomes. A difference in difference approach that compared the CHCs which transitioned from not using it to using it with those that did not use telehealth in two consecutive years also produced null results. However, among rural areas, the odds of better managing the outcomes were greater for certain outcomes. Our study provides limited support that the adoption of telehealth is potentially beneficial in improving certain outcomes in the CHCs setting that are based in rural areas. More specificity in data regarding the nature of telehealth implementation in the CHC setting could bring clarity in these associations.

## 1. Background

Cardio-metabolic syndrome (CMS) consists of a combination of health conditions or outcomes including diabetes mellitus, systemic arterial hypertension, central obesity and hyperlipidemia [1,2,3]. Cardiovascular diseases, resulting from diabetes and hypertension, have been recognized as a global concern attributing to the death of around 18 million people around the world. With over 1.1 billion overweight and 312 million obese adults, this cluster of health conditions has become a global level 5 pandemic [1]. In the United States, these outcomes are recognized as a major public health concern [2], with an estimated prevalence to be over 30% [3].

The management of these measures/outcomes involves reducing, reversing or preventing modifiable risk factors including hypertension, excess weight, obesity, insulin resistance, hyperglycemia, lipid abnormalities, prediabetes and diabetes. [4]. However, public health interventions to effectively deal with these conditions have been complicated [3]. In fact, a recent study suggests that blood glucose health has deteriorated over time [5]. Managing CMS therefore continues to pose a significant challenge to the healthcare system that necessitates fundamental changes in the delivery and maintenance of patient care. Primary care providers, in particular, are in a position that can help address such concerns. For instance, primary care providers screen to identify overweight or obese individuals, and recommend the appropriate weight-loss treatment plans that can prevent or manage diabetes. However, the former’s counseling around weight loss has also declined over time [6]. This is of concern because primary care provider-initiated counseling is associated with an increased desire to lose weight and is associated with a clinically significant weight loss. The latter can also in-turn reduce diabetes risk or the severity of diabetes [7,8].

One of the key strategies that has therefore been identified to manage such outcomes, includes the expansion of telehealth-related services to provide care where people live. Such services may include telemedicine, e-monitoring and text messaging which healthcare systems can promote quality improvement in supporting those with such outcomes [9]. Approaches like these that involve delivering health services at a distance using information and communication technology can enable patients with such conditions to not only seek frequent medical support and guidance, but also, reduce health care costs and promote adherence with the treatment regimens [10,11].

Then, chronic conditions like CMS, partially due to their long duration and slow progression [12], are usually dealt with by primary care providers [13]. Patients’ progress on most of these diseases can be monitored remotely and relapses can be successfully prevented [14], suggesting that the use of telehealth by primary care providers, in the forms of remote patient monitoring and mobile health, has a potential to better manage such conditions. It is also important to note here, that 20% of Americans live in rural and underserved areas and have higher rates of such conditions [15]. Still, there appears to be a dearth of studies that have analyzed the association between telehealth and the cluster of major cardio-metabolic outcomes among such patient groups. There is also a dearth of studies that have looked at any of the latter outcomes across the entire U.S. or at least covering several states, if not all.

The purpose of this study was to analyze the association between organizational level telehealth service provision and the management of CMS-related quality measures within patient populations served by the community health centers (CHCs). The CHCs are nonprofit primary healthcare providers that have been a pivotal part of the healthcare safety net, offering comprehensive prevention and primary care services to uninsured/underinsured populations, and/or in underserved areas [16], and “reducing disparities in healthcare outcomes” [17]. The CHCs are in a unique position to reap the benefits of telehealth. This is not only because they serve the disproportionate number of patients with conditions that require constant monitoring, such as CMS, but also many of the CHC patients often lack a means to travel to the site of care, resulting in care discontinuity and compromised health outcomes. The specific quality measures of interest for this study include the management of hypertension, diabetes, body mass index (BMI), weight, and coronary artery disease. We hypothesize that those with reported telehealth use, as opposed to those without, will be able to better manage the CMS-related quality measures of interest.

## 2. Materials and Methods

### 2.1. Data Source

We used the Uniform Data System (UDS) administrative database for the years 2016–2018. The UDS, a national secondary annual data, is reported to Health Resources and Services Administration by CHCs [17]. A majority of the CHCs operate several care delivery sites; however, UDS data are submitted at the administrative level. The UDS is comprised of CHC patient demographics, insurance types, staffing, scope, and the volume of services CHCs provide, the quality of care, health outcomes and disparities, the number of delivery sites, finances, and electronic health record information.

### 2.2. Study Design

We used organization-level administrative secondary data from the UDS for the years 2016–2018 to analyze the association between telehealth use and the management of myriad cardio-metabolic quality measures, elaborated subsequently. 

### 2.3. Study Sample

The study sample consisted of CHCs that met both federal requirements and received grants under Section 330 of the Public Health Act, which relates to organizations that serve special populations, such as the homeless, low-income and/or underserved. This study captured data on 1367 (year 2016), 1373 (year 2017), and 1362 (year 2018) CHCs.

### 2.4. Variables

The variables were categorized as outcome variables, primary exposure of interest, and other covariates in this effort as follows—(i) Outcomes: The outcomes of interest comprised of the Centers for Medicare and Medicaid Services’ electronic clinical quality measures [18,19]. These were captured in a structured form from the providers’ electronic health record system during the process of patient care [20]. We used managed hypertension (percentage of adult (≥18 years) patients with diagnosed hypertension, whose blood pressure was less than 140/90 mm/Hg (adequate control) during the measurement year), managed diabetes (percentage of adult (≥18 years) patients, with diagnosed diabetes who had hemoglobin A1c greater than 9%), preventive care and screening of body mass index (BMI) (percentage of adult (≥18 years) patients, with BMI that is outside of normal parameters and a documented follow-up plan), weight assessment and counseling for nutrition and physical activity (percentage of children (3–17 years) with a documented BMI percentile and documented counseling for nutrition and physical activity), lipid therapy for coronary artery disease (CAD) (percentage of adult (≥18 years) patients, with a diagnosis of CAD that were prescribed a lipid-lowering therapy), and antiplatelet therapy for ischemic vascular disease (IVD) (percentage of adult (≥18 years) patients, who had a diagnosis of IVD that were taking antiplatelet (aspirin)); (ii) Exposures: The primary independent variable of interest was telehealth use (yes/no). The survey did not consistently gather further information about the nature and scope of its use. The survey did however ascertain in what setting it was used, in the year 2018 of the survey, but since the response to this was not required, the data were limited to conduct any analyses. Other aggregate patient level variables included were race/ethnicity (percentages of Whites, Blacks, Asians, and Hispanics), insurance status (percentages of uninsured, private insurance, Medicare, and Medicaid), percentage of those with poverty below 100 federal poverty level (FPL), location (rural/urban based on the administrative site location and patient zip code); while the administrative location was provided as a binary variable in the dataset, the patient’s zip code-based location was estimated as the percentage of persons living in rural and urban areas; organizational level variables included organization size (number of total patients, number of CHC sites). Further details regarding the study variables are available elsewhere [21].

### 2.5. Analyses

We used descriptive statistics with means and standard deviations of the continuous variables, and frequencies and percentages of categorical variables for presenting the CHCs’ organizational characteristics and CMS-related quality measures. For the regression models, we modeled the outcome as a ratio of patients with managed cardio-metabolic outcomes and total patients with such outcomes. While a percentage could have been obtained for each of these outcomes, and normal distribution could have been assumed, this was not chosen because proportion data were limited within the bounds of 0 and 1 [22]. Moreover, we statistically confirmed that the assumption, that residuals are normally distributed, was violated. A priori identified multivariable-adjusted generalized estimating equations (GEE) [23], accounting for correlations in CHCs nested within states using a exchangeable working correlation matrix (chosen based on the Quasi-likelihood under Independence Model Criterion (QIC) statistic), and with a logit link and binomial distribution were then used to model the outcomes. It should be noted that we encountered convergence issues on using a log link. Finally, we also adjusted our model-based raw *p*-values with multiple testing adjustments and reported both raw and adjusted *p*-values.

Our primary analysis involved analyzing the association between telehealth and management of each of the study outcomes pooled over the study period (i.e., years 2016–2018). Our secondary analyses evaluated the association between telehealth and cardio-metabolic outcomes using a difference in difference (DID) approach, including those stratified by the CHC location. The DID approach investigated the outcome management among those CHCs that transitioned from having no documented telehealth use in 2016 to telehealth use in 2017, in comparison with those that did not report using telehealth in both years. Similar analyses were then conducted for years 2017 and 2018. All the GEEs were adjusted for race/ethnicity, payer mix, location, poverty level, organization size, along with the percentage of patients with specific conditions in that CHC, and also for the ratio of the number of total CHC patient visits/total patients for each outcome. 

All analyses were conducted using SAS statistical software version 9.4 [24].

The current effort was exempt from the Institutional Review Board review as a non-human subject secondary data study.

## 3. Results

Overall, there were 1367, 1373 and 1362 CHCs in the years 2016, 2017 and 2018, respectively. Out of these, respectively, 523, 600, 586 CHCs documented using telehealth in the respective study years. Overall, across the three years, 77% and 72% of the CHCs using telehealth and not using telehealth, respectively, were located in rural administrative areas (Table 1).

When CMS-related outcomes of interest were individually regressed on telehealth use, controlling for the essential confounding variables discussed earlier—with reference to no telehealth use—we did not observe an association between telehealth use and outcome management (Table 2).

Furthermore, secondary analyses using a DID approach were conducted to analyze the association of interest. Table 3 shows that as compared to those CHCs that did not use telehealth in both years 2016 and 2017, those that transitioned from no telehealth use in 2016 to telehealth use in 2017 showed statistically insignificant but greater odds for better managing several conditions. For example, the odds of the better management of hypertension were 7% greater (95% CI: 0.98, 1.17) among CHCs that did report using telehealth in 2017 but not in 2016, with respect to the reference category. A same DID model, for the years 2017 and 2018, showed that those that transitioned from not using telehealth in 2017 to using it in 2018, as compared to the reference, better managed BMI, weight, and ischemic vascular disease with antiplatelet therapy. However, these findings were also not statistically significant. 

When the difference in difference models were additionally stratified by the CHC site location (based on both the administrative site’s location and the patient’s zip code), important differences were revealed (Table 4). While overall with the difference in difference approach stratified by CHC site location there seemed to be a positive association for certain outcomes, this was more pronounced among CHCs located in rural areas. Specifically, among rural CHCs, as identified using the patients’ zip code, those that transitioned from not using telehealth in 2016 to using it in 2017, showed from 5 to 34% greater odds for the better management of the outcomes of interest. However, this was statistically significant only for managing hypertension and BMI. After accounting for multiple testing, only the latter was found to be significant (multiple testing adjusted *p*-value = 0.04) (Table S1).

Table 5 presents the same models for the years 2017 and 2018. The estimates are consistent with those observed in Table 4 and among rural areas as identified using the patients’ zip code, and those that transitioned to using telehealth in 2018 as compared to not using it in 2017, with respect to the reference, were able to better manage certain CMS-related quality measures. The estimates show that among patient zip code based rural areas, those that transitioned as compared to those that did not use telehealth in both the years had a 35% greater odds of better managing weight. However, upon adjusting for multiple testing, this was not significant (adjusted *p*-value = 0.4 (Table S1)).

## 4. Discussion

This research effort analyzed the associations between organizational level telehealth use and the management of CMS-related quality measures among populations served by CHCs. Our primary analysis and secondary DID-based analysis did not reveal any obvious beneficial associations. However, we did find evidence suggestive of a potential positive association between telehealth use and the management of the outcomes of interest among rural areas. This was based on the DID analysis that compared CHCs that transitioned from no documented telehealth use in 2016 to using it in 2017, as opposed to those CHCs that did not document using it for both the years. Similar associations were observed for the years 2017 and 2018 for certain outcomes. It has been argued that the benefits of telehealth provision may indeed be greater in rural areas since remoteness and health care provider shortages may limit access to services [25].

A recent review [11] also confirmed the effectiveness of telemedicine from a global perspective in the care of chronic heart diseases in terms of modality, and quality attributes. The review also noted some limitations like the access to telehealth in rural areas and there not being enough evidence if telehealth indeed was the sole reason for the reported outcomes. On the contrary, another review noted that health outcomes in a large proportion of the studies did not significantly improve with provision of such services, although it did prove to be cost effective from a societal perspective [26].

Specifically, our stratified secondary analysis showed that among patient zip code-based rural areas, CHCs that transitioned from not using telehealth in 2016 to using it 2017 as compared to those that did not use it in both the study years had 34% greater odds (CI: 1.06, 1.70) of managing hypertension specifically. For the years 2017 and 2018 however, these odds were 5% greater as opposed to the reference (0.87, 1.27). Several previous research efforts have also found a positive association between telehealth and hypertension e.g., in a cohort study [27] conducted among 432 adults with prehypertension and hypertension in Taiwan, a decreasing trend in systolic blood pressure was found among 52% of the study cohort. However, others have found no difference among those exposed to telehealth as compared to the control [28]. In the U.S., however, it has been generally evidenced that while hypertension management in the population seems to be improving, other health outcomes like diabetes management continue to lag [29].

Furthermore, the odds of outcomes modeled as a number with BMI management/total (DID with years 2016 and 2017: 1.18 (1.05, 1.30), multiple testing adjusted *p*-value < 0.05; years 2017 and 2018: 1.00 (0.86, 1.16)), and screening for weight assessment/total (1.19 (0.90, 1.59); 1.35 (1.03, 1.77)) were also greater among rural areas. A previous effort [30] that analyzed the effectiveness of videoconferencing technology for delivering comprehensive weight management treatment found that as compared to the control group, the group that received 12 weekly classes through videoconferencing lost weight while the control group gained weight. The mean difference between the groups was −5.5 ± 2.7 kg (−8.0, −3.0). Another randomized controlled clinical trial compared outcomes among 116 recently diagnosed overweight and obese adults. While the intervention group consisted of primary care centers with a telematics platform support, the control group was constantly provided with guidelines to lose weight and follow-up in primary care centers. At the end of the study, weight reduction and an increase in high-density lipoprotein (HDL) cholesterol was observed in both the groups, however, this was more pronounced in the former group than in the controls. As compared to the controls, the former were also four times more likely to achieve a 5% reduction in weight after one year (risk ratio: 4.62; 1.94, 11.0).

Overall, our findings indicate a weak or lack of association between telehealth use in CHCs with the management of CMS-related quality measures. Such weak or lack of association like those observed in the current effort could be due to multiple factors. First, it is possible that in this population the association was indeed weak or null. However, it is also possible that we did not observe the evidence of positive associations between telehealth use and the management of CMS-related quality measures in our primary analysis because our data did not provide specificity on the type of telehealth used. It can also be argued that the use of telehealth in primary care needs to be tailored specifically to suit the needs of both the patients and the primary care providers. Third, the current evidence base and quality of reporting suffers from inconsistencies, which can impact its implementation and effectiveness. In fact, findings from a recent meta-review suggest that the evidence base and quality of reporting, in this rapidly developing field of telehealth-based health services, needs significant improvement in order to inform wider implementation and uptake [5,31].

### Limitations

Beyond asking if telehealth was used or not, no specific questions regarding the nature and scope of its use were asked. While the survey did ask broadly what areas it was used in, this information was only available from a very limited number of CHCs. Hence, this could not be used in the analysis. Then, while the log-binomial model would have been preferred over logit, due to convergence issues, the former could not be used. It can be argued that our estimates would have been closer to null if the former models were used. Lastly, the results of this study may only be generalizable to CHCs with similar characteristics and no other care settings.

## 5. Conclusions

Our study provides only limited support that telehealth adoption by CHCs is beneficial in improving certain cardiometabolic quality measures in primary care settings, specifically in rural areas. There is a need to focus on the science of telehealth implementation in CHCs and perhaps in other pragmatic settings. Future research efforts should also include pragmatic trials that allow evaluating the causal relationship between telehealth use in primary care and cardio-metabolic outcomes that factor the implementation and adoption challenges in CHCs. Economic analyses of telehealth use based on the effect sizes would also be more helpful than simply focusing on statistical significance.

## Figures and Tables

**Table 1:** Descriptive statistics for the study variables by year.

Characteristic	Telehealth	No-Telehealth
Sample Size Per Year		
2016	523	844
2017	600	773
2018	586	776
Summary of Characteristics Across Years		
Cardiometabolic outcomes		
Percentage of patients with managed hypertension, mean (SD)	59.00 (16.50)	56.42 (18.49)
Percentage of patients with controlled diabetes, mean (SD)	31.13 (13.36)	31.75 (14.79)
Percentage of patients that had body mass index preventive care and screening, mean (SD)	55.77 (29.98)	52.08 (30.18)
Percentage of patients that had weight assessment and counseling for nutrition and physical activity, mean (SD)	52.33 (31.51)	47.88 (32.05)
Percentage of patients that had lipid therapy for CAD, mean (SD)	75.92 (18.94)	75.57 (19.25)
Percentage of patients that had antiplatelet therapy for IVD, mean (SD)	74.68 (20.31)	72.71 (21.55)
Provider characteristics		
Number of total patients, mean (SD)	25,038.62 (29,501.47)	16,140.14 (18,127.83)
Number of total service sites, mean (SD)	10.28 (11.26)	6.53 (6.75)
Location, No. (%)		
Urban	548 (22.90)	477 (27.91)
Rural	1845 (77.10)	1232 (72.09)
Patient characteristics		
Race/ethnicity, mean (SD)		
Percentage of Whites patients	44.24 (30.05)	39.53 (30.29)
Percentage of Blacks patients	16.30 (22.00)	20.66 (23.91)
Percentage of Hispanics patients	25.47 (26.21)	27.62 (27.58)
Percentage of Asians patients	2.88 (8.79)	3.36 (9.64)
Insurance status, mean (SD)		
Percentage of patients with private insurance	21.32 (13.00)	18.46 (12.61)
Percentage of patients with Medicare	11.18 (7.19)	10.22 (7.03)
Percentage of patients with Medicaid	42.62 (18.58)	44.43 (19.91)
Percentage of patients who are uninsured/self-pay	24.16 (16.50)	26.13 (19.44)
Percentage of patients who live below 100% federal poverty level, mean (SD)	45.77 (22.98)	48.64 (23.90)
Percentage of patients with hypertension, mean (SD)	17.00 (7.89)	16.94 (7.73)
Percentage of patients with diabetes, mean (SD)	8.50 (3.64)	8.78 (3.95)
Percentage of patients eligible for body mass index screening, mean (SD)	32.21 (19.92)	30.47 (19.94)
Percentage of patients eligible for weight assessment, mean (SD)	16.19 (8.06)	15.25 (8.93)
Percentage of patients eligible for lipid therapy, mean (SD)	1.28 (1.07)	1.24 (1.17)
Percentage of patients eligible for antiplatelet therapy, mean (SD)	2.23 (1.59)	2.16 (1.68)

SD: Standard Deviation; CAD: Coronary Artery Disease; IVD: Ischemic Vascular Disease

**Table 2:** Analyzing the associations between telehealth use and the management of cardio-metabolic syndrome-related quality measures.

Outcomes (CMS-Related Quality Measures)	OR *	(95% CI *)
	Reference: No Telehealth Use	
Number with managed hypertension/total	1.03	(0.98, 1.09)
Number with managed diabetes/total	0.97	(0.92, 1.02)
Number with preventive care and screening of body mass index/total	1.03	(0.99, 1.08)
Number with weight assessment and counseling for nutrition and physical activity/total	1.09	(0.97, 1.23)
Number taking lipid therapy for CAD/total	0.95	(0.83, 1.08)
Number taking antiplatelet therapy for IVD/total	1.00	(0.90, 1.10)

* Generalized estimating equations (GEE) models accounting for community health centers (CHCs) nested within states and adjusted for race/ethnicity, payer mix, location, poverty level, organization size, along with the percentage of patients with specific conditions in that CHC, and the ratio of the number of total CHC patient visits/total patients. Significance level: *p* < 0.05.; CMS: Cardiometabolic Syndrome; OR: Odds Ratio; CI: Confidence Interval; CAD: Coronary Artery Disease; IVD: Ischemic Vascular Disease.

**Table 3:** Analyzing the associations between telehealth use and the management of cardio-metabolic syndrome-related quality measures, employing the difference in difference approach.

Outcomes (CMS-Related Quality Measures)	OR * (95% CI *)			
	(Telehealth Use in 2017−No Telehealth Use in 2016)−(No Telehealth Use in 2017–No Telehealth Use in 2016)		(Telehealth Use in 2018–No Telehealth Use in 2017)–(No Telehealth Use in 2018–No Telehealth Use in 2017)	
Number with managed hypertension/total	1.07	(0.98, 1.17)	0.99	(0.93, 1.05)
Number with managed diabetes/total	1.01	(0.93, 1.10)	0.96	(0.91, 1.02)
Number with preventive care and screening of body mass index/total	1.05	(0.98, 1.11)	1.03	(0.97, 1.10)
Number with weight assessment and counseling for nutrition and physical activity/total	1.11	(0.93, 1.33)	1.08	(0.94, 1.24)
Number taking lipid therapy for CAD/total	1.00	(0.85, 1.19)	0.97	(0.82, 1.14)
Number taking antiplatelet therapy for IVD/total	1.06	(0.92, 1.22)	1.01	(0.88, 1.16)

* GEE models accounting for CHCs nested within states and adjusted for race/ethnicity, payer mix, location, poverty level, organization size, along with the percentage of patients with specific conditions in that CHC, and the ratio of the number of total CHC patient visits/total patients. Significance level: *p* < 0.05; CMS: Cardiometabolic Syndrome; OR: Odds Ratio; CI: Confidence Interval; CAD: Coronary Artery Disease; IVD: Ischemic Vascular Disease.

**Table 4:** Analyzing the associations between telehealth use and the management of cardio-metabolic syndrome-related quality measures stratified by the CHC location, employing the difference in difference approach for the years 2016 and 2017.

	Administrative Location Based				Patient Zip Code Based			
	OR *	(95% CI *)	OR *	(95% CI *)	OR *	(95% CI *)	OR *	(95% CI *)
	Difference in Difference: ((Telehealth Use in 2017–No Telehealth Use in 2016)–(No Telehealth Use in 2017–No Telehealth Use in 2016))							
Outcomes (CMS-related quality measures)	Urban		Rural		Urban		Rural	
Number with managed hypertension/total	1.05	(0.94, 1.18)	1.09	(0.95, 1.25)	1.04	(0.94, 1.13)	1.34	(1.06, 1.70)
Number with managed diabetes/total	1.01	(0.91, 1.12)	1.00	(0.89, 1.13)	1.00	(0.92, 1.09)	1.13	(0.92, 1.38)
Number with preventive care and screening of body mass index total	1.04	(0.96, 1.13)	1.05	(0.97, 1.14)	1.03	(0.96, 1.11)	1.18	(1.05, 1.30)
Number with weight assessment and counseling for nutrition and physical activity/total	1.09	(0.87, 1.36)	1.18	(0.90, 1.55)	1.12	(0.91, 1.36)	1.19	(0.90, 1.59)
Number taking lipid therapy for CAD/total	0.99	(0.80, 1.23)	1.08	(0.86, 1.36)	0.99	(0.82, 1.21)	1.05	(0.77, 1.44)
Number taking antiplatelet therapy for IVD/total	1.10	(0.91, 1.32)	1.04	(0.86, 1.26)	1.03	(0.88, 1.20)	1.32	(0.97, 1.80)

* GEE models stratified by location and accounting for CHCs nested within states and adjusted for race/ethnicity, payer mix, poverty level, organization size, along with the percentage of patients with specific conditions in that CHC, and the ratio of the number of total CHC patient visits/total patients. Significance level: *p* < 0.05; CMS: Cardiometabolic Syndrome; OR: Odds Ratio; CI: Confidence Interval; CAD: Coronary Artery Disease; IVD: Ischemic Vascular Disease.

**Table 5:** Analyzing the associations between telehealth use and the management of cardio-metabolic quality measures stratified by the CHC location, employing a difference in difference approach for the years 2017 and 2018.

	Administrative Location Based				Patient Zip Code Based			
	OR *	(95% CI *)	OR *	(95% CI *)	OR *	(95% CI *)	OR *	(95% CI *)
	Difference in Difference: ((Telehealth Use in 2018–No Telehealth Use in 2017)–(No Telehealth Use in 2018–No Telehealth Use in 2017))							
Outcomes (CMS-related quality measures)	Urban		Rural		Urban		Rural	
Number with managed hypertension/total	0.99	(0.91, 1.07)	0.98	(0.89, 1.09)	0.98	(0.91, 1.04)	1.05	(0.87, 1.27)
Number with managed diabetes/total	0.96	(0.89, 1.03)	0.96	(0.88, 1.04)	0.96	(0.91, 1.03)	0.99	(0.85, 1.17)
Number with preventive care and screening of BMI/total	1.06	(0.97, 1.15)	1.00	(0.91, 1.09)	1.04	(0.97, 1.12)	1.00	(0.86, 1.16)
Number with weight assessment and counseling for nutrition and physical activity/total	1.09	(0.96, 1.32)	1.00	(0.82, 1.22)	1.04	(0.89, 1.21)	1.35	(1.03, 1.77)
Number taking lipid therapy for CAD/total	1.03	(0.83, 1.27)	0.87	(0.68, 1.10)	0.94	(0.79, 1.13)	1.18	(0.87, 1.60)
Number taking antiplatelet therapy for IVD/total	1.11	(0.91, 1.35)	0.85	(0.70, 1.03)	0.99	(0.85, 1.15)	1.14	(0.85, 1.53)

* GEE models stratified by location and accounting for CHCs nested within states and adjusted for race/ethnicity, payer mix, poverty level, organization size, along with the percentage of patients with specific conditions in that CHC, and the ratio of the number of total CHC patient visits/total patients. Significance level: *p* < 0.05; CMS: Cardiometabolic Syndrome; OR: Odds Ratio; CI: Confidence Interval; CAD: Coronary Artery Disease; IVD: Ischemic Vascular Disease.

## Supplementary Materials

The following are available online at https://www.mdpi.com/2227-9032/8/2/165/s1, Table S1: Raw and multivariable adjusted *p*-values for Table 4 and Table 5.
