# Supplementary material for: Telehealth Use among Community Health Centers and Cardio-Metabolic Health Outcomes
Source: Healthcare (Basel). 2020 Jun 10;8(2):165. doi: 10.3390/healthcare8020165 (PMC7348805; doi:10.3390/healthcare8020165)
Supplement: Supplementary file 1 [file healthcare-08-00165-s001.pdf]

Table S1. Raw and multivariable adjusted p-values for table 4 and 5

	**Table 4**								**Table 5**							
	**Zip code: Urban**				**Zip code: Rural**				**Zip code: Urban**				**Zip code: Rural**			
	**[(Telehealth use in 2017 – No telehealth use in 2016) – (No telehealth use in 2017 – No telehealth use in 2016)]**				**[(Telehealth use in 2017 – No telehealth use in 2016) – (No telehealth use in 2017 – No telehealth use in 2016)]**				**[(Telehealth use in 2017 – No telehealth use in 2016) – (No telehealth use in 2017 – No telehealth use in 2016)]**				**[(Telehealth use in 2017 – No telehealth use in 2016) – (No telehealth use in 2017 – No telehealth use in 2016)]**			
	**Raw**	**Stepdown Bonferroni**	**Hochberg**	**False Discovery Rate**	**Raw**	**Stepdown Bonferroni**	**Hochberg**	**False Discovery Rate**	**Raw**	**Stepdown Bonferroni**	**Hochberg**	**False Discovery Rate**	**Raw**	**Stepdown Bonferroni**	**Hochberg**	**False Discovery Rate**
Number with managed hypertension / total	0.533	1.000	0.969	0.800	0.013	0.143	0.143	0.078	0.481	1.000	0.992	0.883	0.618	1.000	0.992	0.883
Number with managed diabetes / total	0.969	1.000	0.969	0.969	0.246	1.000	0.969	0.562	0.247	1.000	0.992	0.883	0.943	1.000	0.992	0.992
Number with preventive care and screening of body mass index / total	0.370	1.000	0.969	0.635	0.003	0.041	0.041	0.041	0.226	1.000	0.992	0.883	0.992	1.000	0.992	0.992
Number with weight assessment and counseling for nutrition and physical	0.281	1.000	0.969	0.562	0.219	1.000	0.969	0.562	0.663	1.000	0.992	0.883	0.032	0.385	0.385	0.385

activity / total																
Number taking lipid therapy for CAD / total	0.956	1.000	0.969	0.969	0.757	1.000	0.969	0.909	0.534	1.000	0.992	0.883	0.296	1.000	0.992	0.883
Number taking antiplatelet therapy for IVD /total	0.731	1.000	0.969	0.909	0.082	0.816	0.816	0.326	0.905	1.000	0.992	0.992	0.387	1.000	0.992	0.883
